# Understanding the Experiences of Patients With Pancreatic Cancer: Quantitative Analysis of the Pancreatic Cancer Action Network Patient Registry

**DOI:** 10.2196/65046

**Published:** 2025-05-26

**Authors:** Allison Rosenzweig, Sydney Rathjens, Kawther Abdilleh, Dennis Ladnier, Fatima Zelada-Arenas, Sudheer Doss, Lynn M Matrisian

**Affiliations:** 1 Pancreatic Cancer Action Network, 2101 Rosecrans Ave, Suite 3200, El Segundo, CA, 90245, United States, 1 310-725-0025

**Keywords:** pancreatic cancer, patient-reported outcomes, patient registry, pain, depression, cancer, patient outcomes, pancreatic, statistical analyses, survey, cancer patient, patient experience, registry, data collection, health status, well-being

## Abstract

**Background:**

The Pancreatic Cancer Action Network (PanCAN) established its Patient Registry to gather real-world data from patients with pancreatic cancer and their caregivers, related to their diagnosis, symptoms and symptom management, treatments, and more. Results from version 2 of the PanCAN Registry are presented here.

**Objective:**

We sought to gather and evaluate patient-reported outcomes data inputted into the PanCAN Patient Registry from December 2020 to January 2024. Statistical analyses were used to identify findings from a relatively small sample size (271 participants, as defined by people who filled out the Basics survey of the PanCAN Registry).

**Methods:**

Participation in the PanCAN Patient Registry was voluntary, and participants filled out an electronic consent form before joining the registry. Participants were identified through the PanCAN Patient Services Help Line or navigated to the registry directly via the PanCAN website. Data analysis took place via bivariate analysis using the chi-square test for categorical variables. Statistical significance was defined as a *P* value of <.05, with *P* values between .05 and .1 considered marginally significant, and *P* values >.1 considered insignificant.

**Results:**

Pain was reported by 186 out of the 207 (89.9%) PanCAN Patient Registry participants who filled out the pain-related questions in the General Assessment survey. We observed a marginally significant (*P*=.06) difference between the reporting of pain by patients aged younger than 65 years (86/92, 93.5%) and those aged 65 years or older (66/78, 84.6%). Depression was also a common condition experienced by patients with pancreatic cancer, with 64/103 (62.1%) indicating that they were experiencing or had experienced depression during the course of their illness. A trend suggested that depression was more frequently reported among the subset of patients who also reported pain (53/80, 66.3%) compared with those who did not report pain (5/13, 38.5%*; P*=.07).

**Conclusions:**

The use of patient-reported outcomes and real-world data for patients with pancreatic cancer has the potential to have direct impact on clinical practice. Through a relatively small sampling of patients, trends were identified that suggest a higher reporting of pain amongst patients in a younger age group as well as concurrence of pain and depression. These findings underscore the importance of a multidisciplinary team of health care professionals addressing patients’ needs beyond the treatment of their cancer.

## Introduction

Cancer registries play a pivotal role in collecting comprehensive data about patients with cancer, which is essential for advancing research and improving patient outcomes. Patient-reported outcomes (PROs) are crucial in this context as they provide direct insights from patients regarding their health status, encompassing physical, mental, and social well-being. Electronic PROs offer an efficient and standardized way to gather this data electronically, enhancing the accuracy and depth of patient information without interpretation by a clinician [1]. Previous studies of patients with advanced cancer suggest that patient-reported symptom monitoring is associated with prolonged survival [2].

The Pancreatic Cancer Action Network (PanCAN) Patient Registry is an online, pancreatic cancer-specific, global registry enabling patients to self-report sociodemographics, characteristics of the disease and its management, and PROs. There have been 2 versions of PanCAN’s Patient Registry, which has been in operation since 2015. Gupta et al [3] explored the usability and usefulness of PROs through data in the PanCAN Patient Registry version 1. This paper [3], which served as a precursor to this study, described the development of the PanCAN Registry and its questions and flow, the user experience, and the application of data generated, emphasizing the value of leveraging PROs to identify trends in diagnosis, treatment, and management of people with pancreatic cancer. The results reported in this analysis are based on the PanCAN Registry version 2. A transition in the vendor managing the PanCAN Registry database technology from the PEER (Platform for Engaging Everyone Responsibly) to LunaDNA as the host occurred in 2020. The LunaDNA platform was built upon the premise that patients owned and had control of their data while having an economic incentive to share it to drive medical research through cryptocurrency [4]. LunaDNA made the decision to close the platform in January 2024 [5].

Both versions of the PanCAN Patient Registry were designed to assist the pancreatic cancer community in understanding the “Right Track” for any patient with pancreatic cancer: right team, right tests, right treatment, and the opportunity to share their data [6]. The primary aims of the PanCAN Patient Registry include: (1) identifying differences between treatment practices and symptom management in community and center-of-excellence settings, (2) identifying hypothesis-generating associations between answers given in survey questionnaires, including molecular data, treatments, family history, care team choices, and patient outcomes, (3) facilitating the gathering of information on the use, effectiveness, and side effects of treatments and remedies, and (4) providing a platform for researchers to add customized modules to answer specific research questions and recruit participants for research. From our experience with the first PanCAN Registry, we learned that many patients and their caregivers are interested in sharing information with researchers that can potentially contribute to better outcomes in the future.

Several scientific meeting abstracts and publications have resulted from the information collected from more than 2000 patients or caregivers who participated in the PanCAN Registry, version 1. Through registry data, we have observed that a concerning proportion of patients (69/205, approximately 34%) were not correctly prescribed pancreatic enzyme replacement therapy based on the recommended dosage and administration of the medication [7]. Even more alarmingly, only 89/205, or about 43% of patients, fully complied with the recommended administration, leading to poorer relief of symptoms and difficulty gaining weight. Another publication explored prediagnosis pain and symptom management, with data suggesting that patients who experience pain before their pancreatic cancer diagnosis had a higher likelihood of being diagnosed with metastatic disease, had more frequent and more intense symptoms, and faced more challenges with pain management throughout their experience with pancreatic cancer [8].

Pancreatic cancer is one of the deadliest cancer diagnoses. Most patients diagnosed with this disease are diagnosed at an advanced stage where cancer has spread from the pancreas to distant parts of the body, resulting in poor survival [9]. The 5-year survival for all stages of disease is currently 13%, the lowest of the major cancers. The aggressive nature of the disease poses a challenge for the collection of survey and PRO information, yet the unmet need demands that all avenues are used, and the patient experience is known and incorporated in the best practices for treatment and care of people with pancreatic cancer. Participation in the PanCAN Registry not only empowers patients and caregivers by involving them directly in research but also enriches the registry with real-world data crucial for understanding the disease and identifying trends that may provide insights into the diagnosis, treatment, and management of pancreatic cancer. PanCAN intends for our Patient Registry to continue to provide valuable information to inform PanCAN and the scientific community of ways to overcome challenges and improve survival for patients with pancreatic cancer for many years to come.

## Methods

### Participants and Enrollment

Participants in the PanCAN Patient Registry were patients with pancreatic cancer or their caregivers, identified through PanCAN’s Patient Services Help Line. Participation was voluntary and required informed consent for the use of their data in research. Patients and caregivers could independently enroll in the PanCAN Registry through the PanCAN website. Upon creating a profile and signing an online informed consent form, participants completed surveys that documented their experiences with pancreatic cancer. Participants completed surveys providing detailed information on diagnosis, symptoms, treatments, complementary medicine regimens, health care decisions, and more.

### Registry Versions and Platform

There have been 2 versions of PanCAN’s Patient Registry. The results reported in this analysis are based on PanCAN Registry version 2, which was open for enrollment from December 2020 through January 2024. The data collected were facilitated by an online data vendor platform called LunaDNA, which housed the PanCAN Registry survey questions for participants to access. The change to version 2 was due to a transition in the vendor managing the PanCAN Registry database technology. Both registry version 1 and version 2 received institutional review board (IRB) approval through Genetic Alliance, and PanCAN updates the IRB annually to maintain registry study protocol compliance. Although PanCAN Registry version 1 and version 2 used different technology platforms, both functioned similarly as patient-facing databases and adhered to PEER requirements determined by Genetic Alliance.

We provide a Checklist for Reporting Results of Internet E-Surveys (CHERRIES) (Checklist 1) that further describes the platform, the development and testing of questions, marketing, data protection, and more [10].

### Survey Development and Data Collection

PanCAN staff worked with LunaDNA, the platform vendor, to transpose the surveys into the proper platform formatting, including branching logic and data extraction. The surveys used in version 2 of the PanCAN Registry were previously developed in version 1 of PanCAN’s Patient Registry and used with occasional changes or updates. These pancreatic cancer-specific surveys were developed and reviewed by experts in the domain and patients affected by pancreatic cancer. The experts included PanCAN staff, oncologists, gastroenterologists, scientists, a dietitian, and a radiation oncologist. The General Assessment survey, previously the Health Assessment survey in PanCAN Registry version 1, included questions derived from the Patient-Reported Outcomes Measurement Information System (PROMIS)-29 validated survey [11,12].

### Data Analysis

Data were extracted from the online LunaDNA-hosted registry (PanCAN Patient Registry version 2). Bivariate analysis was conducted using the chi-square test for categorical variables.Statistical significance was defined as a *P* value of <.05, with *P* values between .05 and .1 considered marginally significant, and *P* values >.1 considered insignificant. Due to the relatively small sample size of this study, a significance level of .1 was used to draw conclusions. While a *P* value of <.05 is a conventional threshold in biomedical research, the use of a .1 threshold is sometimes used in social science research, where increasing sample sizes is not always feasible. The Social Science Statistics calculator includes significance level options of .01, .05, and .10 [13]. While a *P* value of <.05 is a conventional threshold in biomedical research, in this context, the 10-fold difference between a *P* value of .06 and .6 is considered meaningful, and we optimized the significance level for this study as per Mudge et al [14].

Efforts to increase the sample size were not possible due to the unexpected closure of the LunaDNA platform, limiting further recruitment. In addition, publication of results from the PanCAN Registry version 2 is necessary to fulfill patient consent requirements and facilitate further analysis of the data. All user response data collected was deduplicated by identifying unique subject IDs within the deidentified data set. This information was organized in tables to display responses such as demographics, interest in joining the registry, sex, age, and more. All data were manually reviewed and validated by PanCAN staff.

### Survey Participation

Participants could complete up to 7 unique surveys on the PanCAN Registry website, totaling approximately 175 questions if all surveys were completed. Participants were required to complete the Basics survey before accessing additional surveys. The Basics survey gathered information about the person filling out the survey, the patient’s diagnosis and experiences with pancreatic cancer, and high-level information about symptoms, treatments, and reasons for participation. For this study, we defined users as PanCAN Registry participants who had completed at least the Basics survey.

### Technological and Regulatory Framework

The technology, user interface, regulatory requirements, and IRB compliance for the PanCAN Registry platform technology have been previously described [3]. The adherence to IRB requirements for the PanCAN Registry platform technology and the collaboration with LunaDNA to ensure the confidentiality and integrity of the data were described in Gupta et al [3]. All patients that joined the platform to participate in the study had the opportunity to remove their data if they chose. This is why LunaDNA reinforced the use of a sandbox workbench when the protocol was active, and participants were enrolling. However, it was explained to participants that they were not able to remove data that was part of a downloaded research set for publication purposes.

### Ethical Considerations

The Patient Registry received approval for Protocol PCAN001 from the Genetic Alliance IRB on January 19, 2024, as part of its annual review process. Since its launch in 2015, the registry consistently maintained compliance with IRB requirements as determined by the Genetic Alliance.

As described in the informed consent, data privacy and security were central to the registry’s operations. In this agreement with LunaDNA, genomic data (ie, data about an individual’s genes or DNA) and medical or health data (eg, medications, allergies, surveys, health records, and information collected by integrated apps and devices) were referred to as Shared Data. To protect participant privacy, Shared Data were separated from Personal Data, a process referred to as deidentification. Once deidentified, Shared Data were aggregated with data from other participants to create a searchable database designed to support research and discovery while protecting individual privacy.

As outlined in the participation and enrollment section, individuals who wished to join the registry had to create a profile and electronically sign an informed consent form before completing surveys about their pancreatic cancer experiences. Those who chose not to sign the informed consent were not eligible to participate.

Participants may revoke their consent or request deletion of their account at any time, in which case their data will be permanently removed or purged from the database. However, any research already conducted or published using the participant’s data before revocation of consent or data deletion will remain unaffected. Participants did not receive compensation for participation in the patient registry.

## Results

### Demographics of Participants

The demographics of the patient population who participated in version 2 of the PanCAN Patient Registry are shown in Table 1. During the time period analyzed, 272 individuals filled out the basics survey in the LunaDNA-based PanCAN Patient Registry. Of the 191 participants who indicated their age, 1 participant (0.5%) was 11‐15 years old, 13 (6.8%) were aged 25‐44 years, 89 (46.6%) were aged 45‐64 years, and 88 (46.1%) were aged 65 years and above. For the purpose of the analyses described below, we stratified patients as under 65 years (53.9%) or 65 years and older (46.1%).

**Table 1.** Demographics of participants.

Characteristics	Number of participants
Number completing “Basic Survey”^a^	272
Age, years (n=191), n (%)	
<65	103 (53.9%)
11-15	1 (0.5%)
25-44	13 (6.8%)
45-64	89 (46.6%)
≥65	88 (46.1%)
Sex (n=191), n (%)	
Female	96 (50.3%)
Male	95 (49.7%)
Race (multiple options allowed; n=191, responses=210), n (%)	
White	171 (81.4%)
Hispanic, Latino, or Spanish origin	11 (5.2%)
Black or African American	7 (3.3%)
Asian	6 (2.9%)
Middle Eastern or North African	5 (2.4%)
American Indian or Alaskan Native	3 (1.4%)
Central or Southern American Indian	2 (1.0%)
None of these describe me	5 (2.4%)
Stage of cancer at diagnosis (n=272)	
Metastatic	102 (37.5%)
Resectable	75 (27.6%)
Locally advanced	38 (14.0%)
Borderline resectable	35 (12.9%)
I am not sure	22 (8.1%)
Reason for joining the Registry (multiple options allowed, percentage who strongly agree or agree) (n=272 for each question)	
To provide information for researchers and other patients	255 (93.8%)
To learn more about pancreatic cancer	231 (84.9%)
To share information with friends, family, or a doctor	162 (59.6%)
To organize medical records	107 (39.3%)
Someone (eg, family member, doctor) asked me to	44 (16.2%)

a this formed the baseline population of ”Users.”

These 191 participants were evenly distributed by sex, with equal numbers identified as female and male at birth. The population had minimal racial and ethnic diversity, with 191 respondents providing 210 answers (multiple options were allowed). The majority (171/210, 81.4%) identified as White, and 11/210 (5.2%) of participants identified as being of Hispanic, Latino, or Spanish origin, and 7/210 (3.3%) identified as Black or African American.

Of the 272 total participants, 102 (37.5%) were initially diagnosed with metastatic pancreatic cancer, 38 (14%) with locally advanced disease, 35 (12.9%) borderline resectable, and 75 (27.6%) had resectable pancreatic cancer at diagnosis. The remaining 22/272 (8.1%) of respondents were unsure of their stage of disease at diagnosis. It is worth noting that the average distribution of disease stage at diagnosis is 51% metastatic and 14% localized [15], so the patient population in this study was skewed toward earlier stage disease compared with the overall patient population with pancreatic cancer.

Participants were also asked to indicate their reasons for joining the PanCAN Patient Registry. Multiple answers could be selected, and all 272 participants responded to this question. The majority (255, 93.8%) of responses indicated that the participant joined the registry “to provide information for researchers and other patients,” showing a deep sense of altruism. The next most common answer (231, 84.9%) was “to learn more about pancreatic cancer.” A majority (162, 59.6%) of responses indicated that the participant felt the registry would help them “to share information with friends, family, or a doctor.”

### Participants Reporting Pain

Pancreatic cancer and its treatments are known to cause significant pain, typically of the abdominal area and lower back. Participants in the PanCAN Patient Registry were asked several questions pertaining to their experience with pain within the 7 days before their responding to the survey. A total of 7 questions addressed the presence and intensity of pain as well as its interference with day-to-day activities (Supplementary table in Multimedia Appendix 1). For the purpose of this analysis, we stratified the responses to a yes or no response in regard to the participants experiencing pain over the week before filling out the survey (Table 2).

**Table 2.** Responses to pain and depression questions.

Survey item	Number of responses, n (%)	*P* value
Pain		
Reporting pain (n=207)		
Yes	186 (89.9%)	
No	21 (10.1%)	
Reporting pain by sex (n=170)		.58
Male		
Yes	78 (90.7%)	
No	8 (9.3%)	
Female		
Yes	74 (88.1%)	
No	10 (11.9%)	
Reporting pain by age (years; n=170)		.06
<65		
Yes	86 (93.5%)	
No	6 (6.5%)	
≥65		
Yes	66 (84.6%)	
No	12 (15.4%)	
Depression		
Reporting depression (n=103)		
Yes	64 (62.1%)	
No	35 (34%)	
Not sure	4 (3.9%)	
Reporting depression by sex (n=98)		.19
Male		
Yes	26 (56.5%)	
No	18 (39.1%)	
Not sure	2 (4.3%)	
Female		
Yes	36 (69.2%)	
No	14 (26.9%)	
Not sure	2 (3.8%)	
Reporting depression by age (n=91)		.90
<65		
Yes	35 (63.6%)	
No	18 (32.7%)	
Not sure	2 (3.6%)	
≥65		
Yes	22 (61.1%)	
No	12 (33.3%)	
Not sure	2 (5.6%)	
Pain and depression (n=93)		
Those who experienced pain		.07
Depressed		
Yes	53 (66.3%)	
No	24 (30%)	
Not sure	3 (3.8%)	
Those that experienced no pain		
Depressed		
Yes	5 (38.5%)	
No	7 (53.8%)	
Not sure	1 (7.7%)	

Using this methodology, we found that out of 207 respondents, 186 (89.9%) reported pain within the previous 7 days. There was no difference based on sex; approximately 90% of both male and female respondents reported pain.

There was, however, a marginally statistically significant (*P*<.1) difference found in the reporting of pain by age groups, with pain more frequently reported by younger patients. In those aged younger than 65 years, 86/92 (93.5%) reported experiencing some pain over the previous 7 days. A lower percentage (66/78, 84.6%) of individuals aged 65 years and above reported experiencing pain (*P*=.06).

### Participants Reporting Depression

Depression is also frequently experienced by people with pancreatic cancer, as shown in Table 2. For this standalone survey, participants were asked whether they were feeling or had felt “depressed at any time throughout the course of the disease.” There were 103 respondents to this question, and 64 (62.1%) indicated that they were feeling or had felt depressed, 35 (34%) indicated no depression, and 4 (3.9%) were unsure. There were no statistically significant differences in the responses to feeling or had felt depression by sex or by age.

### Concurrence of Pain and Depression

Finally, we were interested in determining the concurrence of pain and depression experienced by individuals who filled out the PanCAN Patient Registry. We hypothesized that those experiencing pain would be more likely to indicate feelings of depression. Indeed, a majority (53/80, 66.3%) of individuals who indicated that they felt pain within 7 days of filling out the survey also said they were experiencing or had experienced feelings of depression. Among individuals who reported no pain, 5/13 (38.5%) answered that they experienced depression. This difference approached statistical significance, with a *P* value of .07.

## Discussion

This study is the first to use data gathered through version 2 of the PanCAN Patient Registry. Although a relatively small dataset, our findings further emphasize the value of PROs in identifying trends in the patient experience and seeking new ways to improve outcomes and quality of life for those facing an extremely challenging diagnosis like pancreatic cancer.

Pain is a well-established frequent symptom experienced by people with pancreatic cancer [8,16,17], and our results showed that nearly 90% of respondents had experienced pain within the previous 7 days of responding to the survey in the PanCAN Registry. Furthermore, we observed a higher frequency of pain reported by younger patients as compared with those aged 65 years and older. Previous analysis of the PanCAN Patient Registry version 1 had shown a higher frequency of prediagnosis pain in younger patients, leading to worse symptom burdens throughout the disease [8]. Other groups have shown a higher prevalence of cancer-related pain being reported by younger versus older patients, across cancer types [18-20]. These results suggest that health care providers pay particular attention to discussing and managing pain experienced by patients who have a younger onset of pancreatic cancer. At the same time, other reports show that patients in an older age group may still experience pain but not report it as frequently as their younger counterparts, showing the need for specialized pain management for all people with cancer [19,21].

Patients with pancreatic cancer tend to experience depression at a higher rate than other cancer types, likely due to physiological changes as well as significant distress caused by diagnosis with an especially deadly type of cancer [22-24]. The concurrence of pain and depression in people with pancreatic cancer [25,26] or other types of cancer and chronic illnesses [27-29] is well-established in the literature and consistent with our findings. This result further emphasizes the urgency of pain management to improve quality of life and mood, as well as the need for routine psychosocial care for people with pancreatic cancer.

The study’s limitations include a small number of participants, limited racial and ethnic diversity, and patients skewed toward an earlier stage of disease compared with the typical distribution of pancreatic cancer diagnoses. Intrinsic to registry-based studies is a bias toward patients with better overall health as well as internet savviness [30]. The answers to the surveys, particularly those specifying the previous 7-day time period rather than the entire course of disease, lead to a bias based upon the timing of the patient’s participation. Finally, we recognize that combining the pain-related questions into yes or no answers removes the granularity of the data, and the full range of patient experiences are not captured.

Overall, our data using version 2 of the PanCAN Patient Registry validate previous findings that pain is more frequently reported in those experiencing pancreatic cancer at a younger age, and that there is a correlation between pain and depression. These results underscore the value of hearing directly from the patients’ perspective and pooling data from patients treated at multiple institutions with varying life and disease experiences. Subsequent research efforts by PanCAN will seek to engage patients of diverse racial and ethnic backgrounds in order to learn more about individual patient experiences and any barriers to high quality and equitable care. Data from both versions of the PanCAN Registry will be made available to the research community by request through a data use agreement [31].

## Supplementary material

10.2196/65046 Multimedia Appendix 1 Supplemental table. Questions pertaining to pain.

10.2196/65046 Checklist 1 Checklist for reporting results of the internet e-surveys: PanCAN patient registry.

## ABBREVIATIONS

IRB: institutional review board
PanCAN: Pancreatic Cancer Action Network
PEER: Platform for Engaging Everyone Responsibly
PROMIS: Patient-Reported Outcomes Measurement Information System
PROs: patient-reported outcomes
