# Supplementary material for: Understanding the Experiences of Patients With Pancreatic Cancer: Quantitative Analysis of the Pancreatic Cancer Action Network Patient Registry
Source: J Particip Med. 2025 May 26;17:e65046. doi: 10.2196/65046 (PMC12149456; doi:10.2196/65046)
Supplement: Multimedia Appendix 1 [file jopm-v17-e65046-s001.docx]

Supplemental table. Questions pertaining to pain.

| **Topic** | **Question** | **Potential answers** | **Stratification for this study** |
| --- | --- | --- | --- |
| Pain | In the past 7 days… How much did pain interfere with your day to day activities? | - Not at all - A little bit - Somewhat - Quite a bit - Very much | - Not at all = no - All other answers = yes |
| Pain | In the past 7 days… How much did pain interfere with work around the home? | - Not at all - A little bit - Somewhat - Quite a bit - Very much | - Not at all = no - All other answers = yes |
| Pain | In the past 7 days… How much did pain interfere with your ability to participate in social activities? | - Not at all - A little bit - Somewhat - Quite a bit - Very much | - Not at all = no - All other answers = yes |
| Pain | In the past 7 days… How much did pain interfere with your household chores? | - Not at all - A little bit - Somewhat - Quite a bit - Very much | - Not at all = no - All other answers = yes |
| Pain | In the past 7 days… How would you rate your pain on average? | 0 (no pain) – 10 (worst pain imaginable) | - 0 = no - 1-10 = yes |
| Gastric Assessment | In the past 7 days… Have you had abdominal discomfort, pain, or cramping? | - Not at all - A little bit - Somewhat - Quite a bit - Very much | - Not at all = no - All other answers = yes |
| Lactose Intolerance | In the past 7 days… I experienced abdominal pain after eating dairy products (such as milk, cheese, ice cream) | - Not at all - A little bit - Somewhat - Quite a bit - Very much | - Not at all = no - All other answers = yes |
