# Supplementary material for: Understanding the Experiences of Patients With Pancreatic Cancer: Quantitative Analysis of the Pancreatic Cancer Action Network Patient Registry
Source: J Particip Med. 2025 May 26;17:e65046. doi: 10.2196/65046 (PMC12149456; doi:10.2196/65046)
Supplement: Checklist 1 [file jopm-v17-e65046-s002.docx]

**Checklist for Reporting Results of Internet E-Surveys (CHERRIES) PanCAN Patient Registry**

| ***Checklist Item*** | ***Explanation*** |
| --- | --- |
| Describe survey design | Participants in the PanCAN Patient Registry were patients with pancreatic cancer, caregivers, or caregivers who previously cared for a loved one. |
| IRB approval | The Patient Registry received institutional review board (IRB) approval through the Genetic Alliance, and PanCAN updates the IRB annually to maintain registry study protocol compliance. |
| Informed consent | Participants filled out an online consent form prior to joining the registry. All participation in the Patient Registry is voluntary. |
| Data protection | LunaDNA put strict parameters in place to adhere to IRB standards and protect patient data privacy when a user entered their information into their profile. All patients that joined the platform to participate in the study had the opportunity to remove their data if they chose. The LunaDNA platform was built upon the premise that patients owned and had control of their data while having an economic incentive to share it to drive medical research through cryptocurrency. |
| Development and testing | The pancreatic cancer-specific surveys used in the Patient Registry were developed and reviewed by experts in the domain and patients affected by pancreatic cancer. The experts included PanCAN staff, oncologists, gastroenterologists, scientists, a dietitian, and a radiation oncologist. |
| Open survey versus closed survey | This was not an open survey. |
| Contact mode | Initial contact of participants was through the PanCAN Patient Services Helpline. The Helpline contacted those who submitted a request for Patient Registry information and provided them with further education. |
| Advertising the survey | Information about the PanCAN Patient Registry was available on pancan.org, or information was offered through the PanCAN Patient Services Helpline. |
| Web/E-mail | This was a web-based survey. Upon creating a profile and signing an online informed consent form, participants completed surveys that documented their experiences with pancreatic cancer. |
| Context | The surveys were hosted on LunaDNA’s registry platform in a custom PanCAN Patient Registry website. Users logged into their created profile to access their survey information. Users were able to see surveys they had previously created or new surveys available to complete. |
| Mandatory/voluntary | The surveys for the Patient Registry were voluntary. |
| Incentives | There were no incentives offered for participation in the PanCAN Patient Registry. |
| Time/Date | PanCAN Registry version 2 was open for enrollment from December 2020 through January 2024. |
| Randomization of items or questionnaires | N/A |
| Adaptive questioning | The PanCAN Patient Registry used adaptive questioning, with some items conditionally displayed based on responses to other items, to reduce the number and complexity of the questions. |
| Number of Items | The total number of items was 175 questions if a participant completed all survey questions within the Patient Registry website. |
| Number of screens (pages) | There were 7 different surveys that participants could complete, formatted in a module setup. |
| Completeness check | Manual completeness checks were done during the data analysis phase. |
| Review step | A ‘Back’ button was available for participant use. |
| Unique site visitor | N/A |
| View rate (Ratio of unique survey visitors/unique site visitors) | N/A |
| Participation rate (Ratio of unique visitors who agreed to participate/unique first survey page visitors) | Participation rate was not calculated. |
| Completion rate (Ratio of users who finished the survey/users who agreed to participate) | Completion rate was not calculated. |
| Cookies used | Cookies were not used. |
| IP check | IP addresses were not collected. |
| Log file analysis | The study did not include a log file analysis. |
| Registration | Participants were able to log in to their user profile and complete surveys. Participants were required to complete the Basics survey before accessing additional surveys. |
| Handling of incomplete questionnaires | Completed surveys and questions were analyzed. |
| Questionnaires submitted with an atypical timestamp | N/A |
| Statistical correction | N/A |

This checklist has been modified from Eysenbach G.’s “Improving the quality of Web surveys: the Checklist for Reporting Results of Internet E-Surveys (CHERRIES)”[1].

1. Eysenbach G. Improving the quality of Web surveys: the Checklist for Reporting Results of Internet E-Surveys (CHERRIES). J Med Internet Res. 2004 Sep 29;6(3):e34. PMID: 15471760. doi: 10.2196/jmir.6.3.e34.
